# SARS-CoV-2 Spike Protein-Induced Interleukin 6 Signaling Is Blocked by a Plant-Produced Anti-Interleukin 6 Receptor Monoclonal Antibody

**DOI:** 10.3390/vaccines9111365

**Published:** 2021-11-20

**Authors:** Collin Jugler, Haiyan Sun, Qiang Chen

**Affiliations:** 1 The Biodesign Institute, Arizona State University, Tempe, AZ 85287, USA; Collin.Jugler@asu.edu (C.J.); haiyan.sun@asu.edu (H.S.); 2 School of Life Sciences, Arizona State University, Tempe, AZ 85287, USA

**Keywords:** SARS-CoV-2, monoclonal antibody (mAb), COVID-19, interleukin 6 (IL-6), IL-6 receptor (IL-6R), cytokine storm, spike protein (S), sarilumab, plant-made therapeutics, plant-made antibody

## Abstract

Severe acute respiratory syndrome coronavirus 2 (SARS-CoV-2), the causative agent of the current COVID-19 pandemic, has caused more than 4.5 million deaths worldwide. Severe and fatal cases of COVID-19 are often associated with increased proinflammatory cytokine levels including interleukin 6 (IL-6) and acute respiratory distress syndrome. In this study, we explored the feasibility of using plants to produce an anti-IL-6 receptor (IL-6R) monoclonal antibody (mAb) and examined its utility in reducing IL-6 signaling in an in vitro model, which simulates IL-6 induction during SARS-CoV-2 infection. The anti-IL6R mAb (IL6RmAb) was quickly expressed and correctly assembled in *Nicotiana benthamiana* leaves. Plant-produced IL6RmAb (pIL6RmAb) could be enriched to homogeneity by a simple purification scheme. Furthermore, pIL6RmAb was shown to effectively inhibit IL-6 signaling in a cell-based model system. Notably, pIL6RmAb also suppressed IL-6 signaling that was induced by the exposure of human peripheral blood mononuclear cells to the spike protein of SARS-CoV-2. This is the first report of a plant-made anti-IL-6R mAb and its activity against SARS-CoV-2-related cytokine signaling. This study demonstrates the capacity of plants for producing functionally active mAbs that block cytokine signaling and implies their potential efficacy to curb cytokine storm in COVID-19 patients.

## 1. Introduction

Severe acute respiratory syndrome coronavirus 2 (SARS-CoV-2) is the causal agent of coronavirus disease 2019 (COVID-19) and has infected over 220 million people, causing more than 5.1 million deaths globally since its emergence [1]. Symptoms vary widely between individuals, from asymptomatic infection to mild, self-limiting symptoms to pneumonia-like disease that may progress to acute respiratory distress syndrome (ARDS), hyperinflammation, viral sepsis, multi-organ failure and death [2,3]. A characteristic seen in severe cases of COVID-19 appears to be a dysregulated immune response, resulting in cytokine storm and immunopathology [4]. This indicates that systemic inflammation is correlated with more severe states of disease [4]. Of interest, increased proinflammatory cytokine levels have a strong correlation with severe symptoms and higher interleukin 6 (IL-6) levels are associated with non-survivors and patients needing mechanical ventilation [4,5,6]. It has been shown that SARS-CoV-2 spike (S) and nucleocapsid proteins alone can induce production of IL-6 in monocytes and macrophages, and such IL-6 upregulation may be a trigger that initiates the dysregulated immune response in some COVID-19 patients [7]. While the underlying mechanisms are still unclear, emerging evidence suggests that innate immune responses triggered by different viral components and mediated through Toll-like receptors (TLRs) are partially responsible in driving immune dysregulation [8,9,10,11]. In silico studies have predicted that SARS-CoV-2 interacts with host cells through several TLRs [10] and it has now been established that the S protein can indeed trigger a proinflammatory response through TLR4, specifically inducing production of IL1-β and IL-6 mRNA [11]. Thus, controlling the inflammatory response triggered by SARS-CoV-2 or its viral components in a timely and balanced fashion may prevent the development of severe disease and benefit those who are at risk of suffering severe COVID-19 symptoms.

One approach to curb the hyperinflammatory response involves the inhibition of IL-6 signaling. IL-6 signaling occurs through the secreted protein’s interaction with the IL-6 receptor (IL-6R), which is present in membrane-bound form on hepatocytes, monocytes and lymphocytes, and the soluble form that arises from the proteolytic cleavage and alternative splicing of IL-6R [12,13]. Once bound to IL-6R, the IL-6/IL-6R complex binds with the transmembrane gp130 that acts as the signal transducer and is ubiquitous across many cell types and tissues. This signaling ultimately results in activation of JAK-MAPK and JAK-STAT signaling pathways that can activate either proinflammatory or anti-inflammatory effects, depending on whether IL-6R is in its membrane-bound or soluble form [12]. This signaling can be blocked by monoclonal antibodies (mAbs), such as tocilizumab or sarilumab, both of which bind directly to IL-6R, disrupting IL-6 binding. Both of these mAbs are approved for treating rheumatoid arthritis (RA) in the United States and are currently under evaluation for other inflammatory diseases as well [13,14]. Given the approval for RA treatment, there is potential for off-label use of these mAbs for treating severe COVID-19 where circulating IL-6 levels are high.

In recent years, increasing evidence supports the use of plants as a feasible alternative to mammalian cell cultures for mAb development and production due to advantages of low cost, low risk of contamination by animal pathogens, readiness of scale-up production, and glycan modulation [15]. For example, mAbs produced in *Nicotiana benthamiana* have been examined as therapy to treat various pathogenic viruses including flaviviruses [16,17], alphavirus [18], and retrovirus [19] in animal models, as well as Ebola virus in human patients [20]. Furthermore, the robust and rapid nature of plant transient expression [21,22] allows the production of mAbs at unprecedented speed, which can be explored to quickly produce mAbs in large quantities to control the current COVID-19 and future potential pandemics.

Here, we aimed to produce an anti-IL6R mAb (IL6RmAb) in glycoengineered plants and evaluate its efficacy in blocking SARS-CoV-2 S protein-induced IL-6 signaling. IL6RmAb can be quickly expressed in *N. benthamiana* leaves within one week after gene delivery and the plant-made IL6RmAb (pIL6RmAb) is correctly assembled. pIL6RmAb binds to IL-6R with high affinity and is functional in suppressing IL-6 signaling in a cell-based model system. Notably, pIL6RmAb also efficiently inhibits IL-6 signaling mediated by IL-6 that is induced from SARS-CoV-2 S protein exposure. Plant-produced anti-IL6R mAbs may help contribute to the further development of affordable therapeutics for SARS-CoV-2.

## 2. Materials and Methods

### 2.1. Materials

Reagents for phosphate-buffered saline, sodium L-ascorbate, sodium carbonate, sodium bicarbonate, ammonium persulfate, tetramethylenediamine (TEMED), and lipopolysaccharide (LPS) were purchased from Sigma-Aldrich (St. Louis, MO, USA). 96-well polystyrene plates for ELISAs were purchased from Corning (Kennebunk, ME, USA). Secondary antibodies for ELISAs and Western blots were purchased from SouthernBiotech (Homewood, AL, USA). The KPL 3,3′,5,5′-tetramethylbenzidine (TMB) peroxidase substrate kit for ELISA was purchased from SeraCare (Milford, MA, USA). Ethylenediaminetetraaccetic acid (EDTA), Tween-20, Tris-base, and sodium dodecyl sulfate (SDS) were purchased from Fisher Scientific (Fair Lawn, NJ, USA). 4–20% gradient gels, 30% (29:1) acrylamide/bis solution for SDS-PAGE and polyvinylidene fluoride (PVDF) membrane for Western blots were purchased from Bio-Rad (Hercules, CA, USA). Coomassie Brilliant Blue R-250 and OmniPur^®^ phenylmethylsulfonyl fluoride (PMSF) were purchased from EMD Chemicals (Gibbstown, NJ, USA). Electrophoresis grade glycine was purchased from MP Biomedicals LLC (Solon, OH, USA). Millipore Express PLUS 0.22 µm vacuum membranes and Pierce ECL Western blotting substrate were purchased from Thermo Scientific (Rockford, IL, USA). MabSelect Protein A resin was purchased from GE Healthcare Life Sciences, now known as Cytiva (Upsala, Sweden). Recombinant human IL-6 was purchased from PeproTech (Rocky Hill, NJ, USA). The IL-6 Bioassay (includes IL-6 Bioassay cells, Fetal Bovine Serum (FBS), RPMI 1640 Medium, Bio-Glo™ Luciferase Assay Buffer, and Bio-Glo™ Luciferase Assay Substrate) was purchased from Promega (Madison, WI, USA). Recombinant SARS-CoV-2 S1 protein (32-190005) was purchased from Abeomics (San Diego, CA, USA). Human buffy coats were purchased from BioIVT (Westbury, NY, USA). Ficoll-Paque PLUS was purchased from Global Life Sciences Solutions USA LLC (Cytiva) (Marlborough, MA, USA). Human IL-6 ELISA MAX™ Deluxe Kits were purchased from Biolegend^®^ (San Diego, CA, USA).

### 2.2. Agroinfiltration of N. benthamiana

The amino acid sequence of sarilumab was obtained from the KEGG Drug Database (https://www.genome.jp/dbget-bin/www_bget?dr:D10161, accessed 18 June 2020) and the heavy chain (HC) and light chain (LC) were used to generate coding sequences for plant-based expression using Gene Designer 2.0 [23]. Designed gene fragments were synthesized by Integrated DNA Technologies (Coralville, IA, USA). The synthesized HC and LC genes were then cloned into an optimized geminivirus bean yellow dwarf virus-based expression vector, transformed into *Agrobacterium tumefaciens* EHA105 cells by electroporation and verified by PCR as described previously [24]. The verified strain was grown overnight in YenB medium with kanamycin and rifampicin and then used to infiltrate 5–7-week-old *N. benthamiana* plant leaves as described [25]. Leaf tissue was harvested at 5 days post infiltration.

### 2.3. Protein Extraction and Purification

Plant leaves expressing IL6RmAb were homogenized in an extraction buffer consisting of phosphate-buffered saline pH 5.2 with 10 mg/mL of Na-L-ascorbate, 1 mM EDTA, and 2 mM PMSF. The homogenate was clarified by centrifugation, followed by an overnight incubation at pH 5.2, and further centrifugations. The total protein extract was filtered through a 0.22 µm filter and pIL6RmAb was purified by Protein A affinity chromatography as described previously [26].

### 2.4. SDS-PAGE and Western Blot

Purified pIL6RmAb was separated under reducing and non-reducing conditions on a 4–20% gradient gel alongside a human IgG control and stained with Coomassie Brilliant Blue R-250 as described previously [27]. The purity of pIL6RmAb was measured by quantitating Coomassie blue-stained bands on SDS-PAGE with a densitometer [26,28]. For western blots, protein samples were separated on 12% or 4–20% gels under reducing or non-reducing conditions, respectively. After transfer to PVDF membrane, proteins samples were probed with goat anti-human kappa-HRP or gamma-HRP. Blots were then developed with Pierce ECL Western blotting substrate according to manufacturer’s instructions and images taken with the ImageQuant LAS 4000 imager and associated software.

### 2.5. ELISA

pIL6RmAb expression was quantitated by ELISA that detected the assembled form of mAbs with both HC and LC, as described [28]. Briefly, plates were coated with a goat anti-human gamma HC antibody (Southern Biotech). After incubation with the plant protein extract, an HRP-conjugated anti-human-kappa LC antibody (Southern Biotech) was used for detection. A plant produced mAb with human IgG1 CH and kappa CL (E16) [28] was used as a reference standard.

The ELISA for measuring the binding of pIL6RmAb to IL-6R was performed as described [29]. Human IL-6R (2 µg/mL, Sino Biological) was immobilized on microtiter plates. An HRP-conjugated anti-human-gamma HC antibody was used as the detection antibody. The plates were developed with tetramethylbenzidine substrate (KPL Inc. Gaithersburg, MD, USA). A generic human IgG was used as an IgG isotype negative control. Experiments were performed at least two times with technical quadruplicate for each sample. The binding data were analyzed with GraphPad Prism software. KD was determined by non-linear regression analysis using a one-site binding model.

### 2.6. IL-6 Signaling Inhibition

The bioactivity of pIL6RmAb was analyzed with the IL-6 Bioassay from Promega [30], following the manufacturer’s instructions. Briefly, the human cells containing a luciferase reporter under the control of an IL-6-dependent response element were plated in an opaque 96-well plate. Serial dilutions of pIL6RmAb or a generic human IgG (isotype IgG negative control) were performed and added to wells containing cells and incubated for 20 min. Recombinant IL-6 was then added to the wells and incubated for 6 h to allow for IL-6 signaling. Bio-Glo™ reagent was then added to the plate and luminescence was measured using a luminescent plate reader. Fold-induction (ratio of sample luminescence to control luminescence) was calculated and plotted versus pIL6RmAb concentration using a 4PL curve in GraphPad Prism 9.2, by which the 50% inhibitory concentration (IC_50_) was calculated.

### 2.7. SARS-CoV-2 S Protein Stimulation of PBMCs

Human PBMCs were isolated from buffy coats as described [31]. Either fresh or cryo-preserved, PBMCs were cultured in complete RPMI-1640 media, supplemented with streptomycin and penicillin for 24 h. Cells were counted, split evenly (2–7 × 10^6^) into a 12-well plate and recombinant SARS-CoV-2 spike protein was added to the cells at a concentration of 1 µg/mL. In parallel, LPS was added to the cells at a concentration of 200 ng/mL as a positive control for IL-6 stimulation in PBMCs. The PBMC supernatant was collected after 72 h, and the IL-6 concentration was determined by ELISA. A student’s *t*-test (paired) was used to for statistical analysis in GraphPad Prism 9.2.

### 2.8. Inhibition of SARS-CoV-2 S Protein-Induced IL-6 Signaling

The IL-6 Bioassay cells from Promega were used similar to the manufacturer’s instructions to assess the capacity of pIL6RmAb to inhibit IL-6 signaling in our SARS-CoV-2 S Protein, PBMC model in vitro. Briefly, 2.5 × 10^5^ cells were added to each assay well and cells were allowed to adhere to an opaque 96-well plate overnight at 37 °C with 5% CO_2_. The following day, media was aspirated, and antibody dilutions at 2× concentrations were incubated with adhered cells for 20 min at 37 °C with 5% CO_2_ before addition of PBMC supernatant to bring the antibody concentration to 1X and the IL-6 concentration in the supernatant to 0.5× of the concentration determined by ELISA. The antibody/supernatant/cell mixture was incubated for 24 h followed by addition of the Bio-Glo™ reagent and luminescence reading with a luminescent plate reader. Fold induction of luciferase was calculated and transformed into percent luciferase signal relative to the untreated supernatant control. One-way ANOVA was used for statistical analysis in GraphPad Prism 9.2.

## 3. Results

### 3.1. Expression and Characterization of IL6RmAb Produced in N. benthamiana

**Figure 1:**
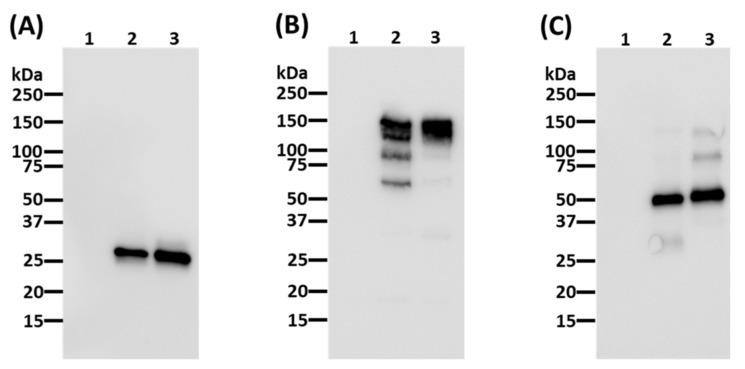
Western blot analysis of plant-produced IL6RmAb (pIL6RmAb). IL6RmAb was purified from *N. benthamiana* leaves and separated on SDS-PAGE gels under reducing (**A**,**C**) or non-reducing (**B**) conditions. After transferring the proteins to onto PVDF membranes, a house radish peroxidase (HRP)-conjugated goat anti-human kappa chain antibody (**A**,**B**) or an HRP-conjugated goat anti-human gamma chain antibody (**C**) were used to detect light chain or heavy chain, respectively. Lane 1, protein sample (~25 µg) from non-infiltrated leaves; Lane 2, protein sample (1 µg) from leaves infiltrated with IL6RmAb-expressing construct; Lane 3, protein sample of purified human IgG (1 µg).

**Figure 2:**
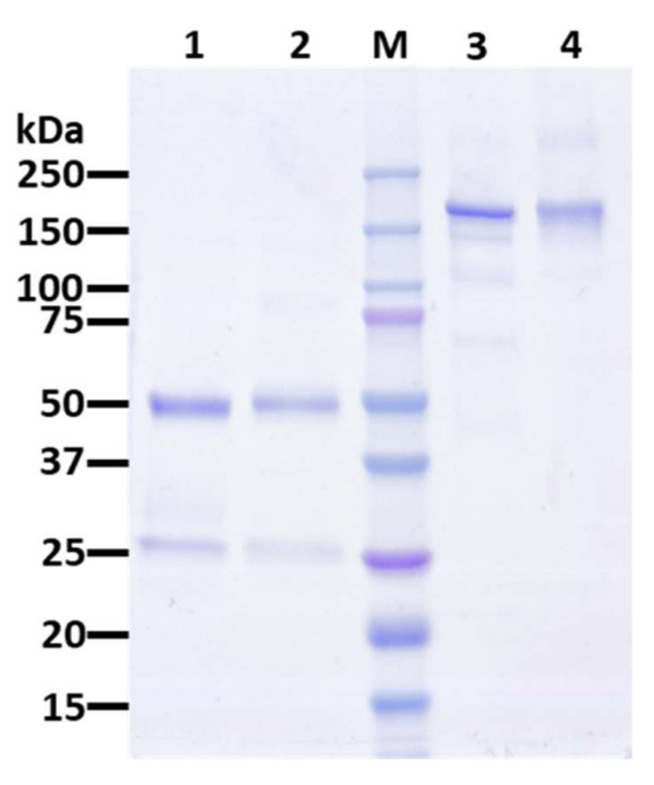
Purification of pIL6RmAb from *N. benthamiana* plants. Protein A affinity column-purified pIL6RmAb from leaves was analyzed on a 4–20% gradient SDS-PAGE gel under reducing (Lane 1) or non-reducing (Lane 3) conditions and visualized with Coomassie staining analysis. Lanes 1 and 3, Protein A-purified pIL6RmAb (2.5 µg); Lanes 2 and 4: human IgG as an isotype reference standard (2.5 µg); M: protein molecular weight marker; One representative of several independent experiments is shown.

Agroinfiltration was used to transiently express the full heterodimeric IgG_1_ pIL6RmAb in *N. benthamiana* through the use of a geminivirus bean yellow dwarf virus-based vector system [21,22,32,33,34]. Expression of the fully assembled pIL6RmAb was monitored by Western blot analysis. Membranes probed for human kappa light chain (Figure 1A,B) and human gamma heavy chain (Figure 1C) show that both the light and heavy chain of the mAb are indeed expressed at the expected sizes and that the full heterotetramer of ~150 kDa is assembled correctly in the plants (Figure 1B). The expression level of IL6RmAb was estimated by ELISA and shown to be 55.95 µg/g fresh leaf weight (FLW) (Figure S1). This level of expression is higher than that previously reported for mAbs against SARS-CoV-2 spike protein produced in plants using the same plant expression vector [35]. The expression level can be improved by using newer versions of the vector that have been optimized for expressing mAbs in plants [36]. Plant-made IL6RmAb (pIL6RmAb) was isolated from soluble plant leaf protein extract and purified through Protein A affinity chromatography prior to further biochemical characterization [37]. As shown in Figure 2, pIL6RmAb was purified to high degree of homogeneity (~98%), comparable to typical, commercially available human IgG.

### 3.2. IL-6R Binding Specificity and Affinity of pIL6RmAb

**Figure 3:**
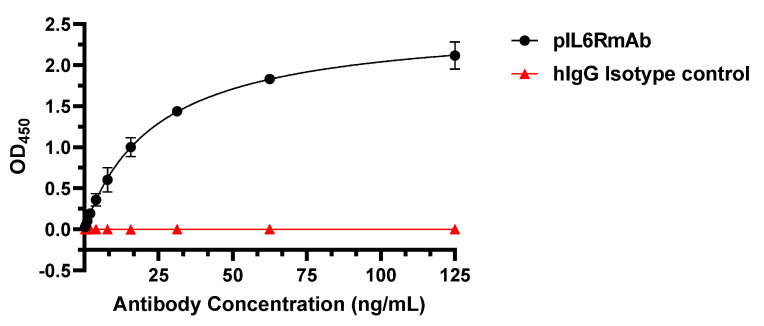
Specific binding of pIL6RmAb to IL-6R. pIL6RmAb was serially diluted and incubated with IL-6R coated on microtiter plate. After incubation, pIL6RmAb bound to IL-6R was detected with an HRP-conjugated anti-human-gamma HC antibody. Dilutions of a generic human IgG (hIgG) were used as the IgG isotype negative control. The OD_450_ values (mean ± SD) from two independent experiments are presented.

To confirm the authenticity and proper folding of pIL6RmAb, the specificity and affinity of pIL6RmAb in binding human IL-6R was assessed by ELISA in which IL-6R was immobilized to microtiter plate. As shown in Figure 3, pIL6RmAb bound IL-6R specifically with high affinity (KD = 0.083 nM) but did not show any binding to the human IgG isotype negative control. This binding affinity is comparable to the KD value reported for the mammalian cell-produced sarilumab (0.062 nM) [38]. Together, these results demonstrated that pIL6RmAb retained IL-6R binding specificity and affinity that are comparable to sarilumab.

### 3.3. IL-6 Signaling Inhibition In Vitro

**Figure 4:**
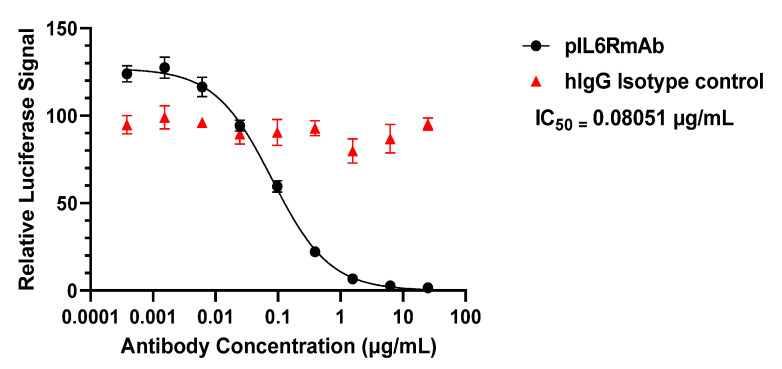
In vitro inhibition of IL-6 signaling by pIL6RmAb. Serial dilutions of pIL6RmAb or the human IgG isotype negative control antibody (hIgG) were incubated with cells containing luciferase as the downstream reporter of IL-6/IL-6R signaling, prior to exposure to recombinant human IL-6. After incubation, luciferase-detecting reagent was added and the bioluminescent signal was measured. The ratio of pIL6RmAb-treated signal to no antibody-treated signal was plotted versus antibody concentration to elucidate the inhibitory activity of antibodies. IC_50_ was calculated using GraphPad Prism 9.2. Two independent experiments were performed, and error bars represent standard error of the mean.

After verifying the purity, assembly, and IL-6R binding affinity of pIL6RmAb, the inhibitory activity of the mAb was analyzed. We used a well-established in vitro cell-based assay in which a luciferase reporter allows for the quantification of IL-6 signaling [38,39]. An inhibitory curve was obtained with serial dilutions of pIL6RmAb and an IC_50_ of 0.081 µg/mL (~0.54 nM) was calculated upon incubation with 25 ng/mL (~1.19 nM) of recombinant human IL-6, while human IgG (isotype IgG negative control) displayed no inhibition of IL-6 signaling (Figure 4). The IC_50_ value of pIL6RmAb is comparable to those of mammalian cell-produced sarilumab obtained by the same assay [38,39,40]. Thus, pIL6RmAb was confirmed to retain its binding to human IL-6R and inhibit the signaling of IL-6 in a predefined in vitro system.

### 3.4. Peripheral Blood Mononuclear Cell (PBMC) Stimulation and Inhibition of Signaling Generated from SARS-CoV-2 Spike Protein-Induced IL-6

**Figure 5:**
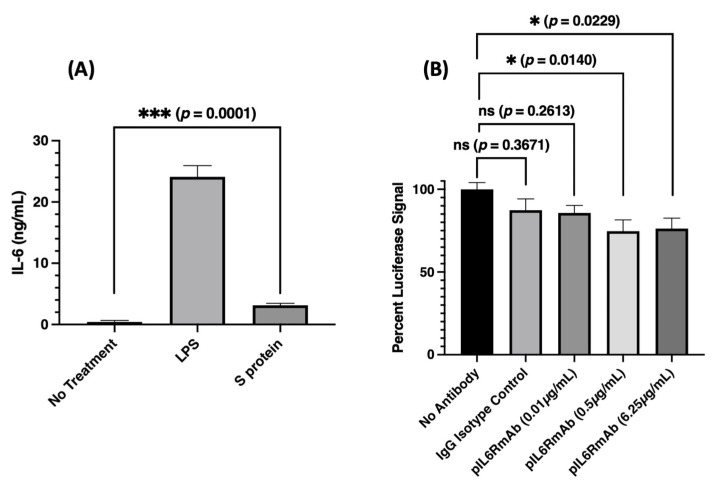
SARS-CoV-2 spike-protein stimulation of human PBMCs and inhibition of spike-protein-induced IL-6 signaling. (**A**) PBMCs were incubated for 72 h with LPS (200 ng/mL, stimulation positive control) or recombinant SARS-CoV-2 S protein (1 µg/mL). The supernatant was collected, and IL-6 concentration was determined by ELISA. (**B**) Luciferase reporter cells were pre-incubated with various concentrations of pIL6RmAb or an IgG isotype antibody negative control (6.25 µg/mL). The supernatant from S protein-stimulated PBMCs was then added to the cells. After 24 h of incubation, Luciferase-detecting reagent was added and the bioluminescent signal was measured. The percentage of luciferase signal relative to no antibody treatment is shown. Student’s t-test and one-way ANOVA were used for statistical analysis. At least three independent experiments were performed, and error bars represent standard error of the mean. *** indicates *p* values < 0.001 of IL-6 secretion by the S protein treated PBMCs compared to that of no treatment negative control. * and ns (not significant) indicate *p* values < 0.05 and > 0.05, respectively, of pIL6RmAb-treated samples compared to that of no treatment negative control.

Once the biological activity of pIL6RmAb was verified with the In vitro IL-6 signaling assay, we tested our hypothesis that the plant-made mAb may have therapeutic potential for reducing SARS-CoV-2-induced inflammation. To this end, it has been reported that SARS-CoV-2 S protein can directly induce production of inflammatory cytokines in monocytes [7]. Indeed, when we incubated PBMCs with recombinant S protein for 72 h, we observed a significant induction of secreted IL-6 from PBMCs (Figure 5A) (*p* = 0.0001 compared to no treatment). We then investigated if pIL6RmAb can inhibit the signaling of SARS-CoV-2 S protein-induced IL-6. When the S protein-stimulated PBMC supernatant with elevated IL-6 was incubated with the luciferase reporter cell line in the presence of various mAbs, no significant inhibition of reporter activity was observed for the isotype IgG negative control (*p* = 0.3671). However, a significant decrease in luciferase activity was detected for pIL6RmAb at concentrations of 0.5 µg/mL and 6.25 µg/mL (*p* = 0.0140 and 0.0229, respectively), but not the 0.01 µg/mL treatment (*p* = 0.2613) (Figure 5B). This data provides In vitro evidence that supports the potential therapeutic use of a plant-made anti-IL6R mAb for reducing SARS-CoV-2-induced, IL-6-driven inflammation.

## 4. Discussion

This is the first report of an anti-cytokine receptor mAb being produced in plants. The IL6RmAb was quickly expressed in *N. benthamiana* plants and assembled correctly with minimal impurities probably due to degradation of the heterotetramer during antibody isolation. Further optimization of the protein purification procedure such as using protease inhibitors and lower temperatures may address the degradation issue. pIL6mAb exhibited similar binding specificity and affinity to human IL-6R as sarilumab, confirming its identity and authenticity. Assessing the biological activity of any mAb for therapeutic treatment is essential and the use of reporter assays provide more relevant information about biological activity over binding data alone. Thus, we subsequently analyzed the bioactivity of the mAb in vitro. Indeed, the IC_50_ obtained with pIL6RmAb with the use of a luciferase-based reporter assay is comparable to other studies [38,39,40], indicating that the plant-made version of the mAb indeed retains its functionality and has therapeutic potential.

Furthermore, when we re-created a SARS-CoV-2-induced immune response In vitro with PBMCs, pIL6RmAb demonstrated a significant capacity to inhibit IL-6 or possibly other potential IL-6 family cytokine signaling from the supernatant of S protein-stimulated PBMCs. However, the inhibitory potency of pIL6RmAb to S protein-induced IL-6 signaling is not as high as we predicted based on the results of pIL6RmAb inhibition on the signaling of pure recombinant IL-6. Specifically, a luciferase signal equivalent to 74.7% and 76.3% of the non-treated control is still observed after 24 h at pIL6RmAb concentrations of 0.5 µg/mL and 6.25 µg/mL, respectively. However, if the In vitro reporter assay we used exclusively measures IL-6/IL-6R signaling, we expect these two concentrations of pIL6RmAb to inhibit ~80% and >95% of the luciferase signal, based on our inhibition curve obtained with recombinant IL-6 in Figure 4. Nevertheless, the luciferase assay used in this study does not exclusively report IL6/IL6R signaling as it uses the gp130 and the STAT3 signaling pathway, which is shared by other IL-6 family cytokines [41]. Indeed, evidence of cross-signaling with other cytokines has been reported for this reporter system [30]. In addition, the response induced from S protein exposure to PBMCs is complicated and many transcriptional and translational changes occur quite rapidly including the secretion of immune modulators other than IL-6 [7]. Thus, other IL-6 family cytokines induced by S protein may be responsible for the observed residual luciferase signal at concentrations of pIL6RmAb that should have almost eliminated the signal if it was solely induced by IL6. The relevance of the observed cross signaling in the reporter system to hyperinflammation during COVID-19 infection is uncertain. However, it is likely other cytokines also contribute to the induction of cytokine storm. Thus, it can be concluded that although pIL6RmAb indeed reduces the IL-6/IL-6R signaling, inhibiting the signaling of other cytokines may be also needed to fully stop the initiation of the cytokine storm. While animal models with humanized IL-6 and IL-6R that recapitulate human disease of COVID-19 are under development, future therapeutic experiments in these animal models are absolutely necessary to determine the clinical benefit of pIL6RmAb in reducing IL-6 signaling for cytokine release syndrome-driven diseases, particularly in the context of SARS-CoV-2 infection.

Normal human serum levels of IL-6 are in the range of 1–10 pg/mL, while levels seen in severely ill COVID-19 patients are on average of 36.7 pg/mL [42]. One retrospective study determined that an IL-6 concentration greater than 24 pg/mL after diagnosis was predictive of hypoxemia, leading to hospitalization and severe disease among elderly patients in a long term care facility [43]. Although this level is much lower than that reported with ARDS, sepsis, or cytokine release syndrome (CRS) associated with other conditions, there is high variability between COVID-19 patients and much remains to be discovered about the immunobiology of SARS-CoV-2 [42,44]. Nevertheless, studies have been performed to investigate the possible benefit of IL-6 antagonists in COVID-19 patients with serious symptoms. These studies include several clinical trials to evaluate the off-label use of anti-IL-6R antibodies (tocilizumab or sarilumab) to treat COVID-19. One trial using either tocilizumab or sarilumab found the use of the IL-6R mAbs to be beneficial for critically ill patients within 24 h of starting organ support, improving clinical outcome and reducing mortality [45]. In contrast, a study analyzing the use of tocilizumab on patients with severe COVID-19 pneumonia showed no statistically significant benefit [46]. Similarly, a phase 3 trial in COVID-19 patients receiving supplemental oxygen also did not reveal significant efficacy of the sarilumab intervention. However, the largest trial to date utilizing tocilizumab (RECOVERY; ClinicalTrial.gov [NCT04381936]), showed that along with the use of corticosteroids, the mAb treatment improves clinical outcomes and survival [47]. Differences in study design, definitions of severe and critical disease states, and enrollment of patients at varying time points through disease progression likely influenced the inconsistent outcomes of these studies. Overall, the published results highlight the importance of timing of therapeutic delivery when treating critically ill COVID-19 patients, particularly with treatments that are modulating the immune response, since there is likely a threshold for therapeutic benefit. Therefore, more studies are needed evaluating the outcome of interrupted IL-6 signaling in COVID-19 disease progression. The rapid production of pIL6RmAb indicates that plant-made anti-IL-6R mAbs may expedite the elucidation of the beneficial timing of IL-6R antagonists.

Plants are a viable option for production of human biological therapeutics, especially mAbs [17,18,48]. The hallmark benefits of plant-based production are lower upstream costs, simple scaling for commercial manufacturing, and requiring relatively inexpensive facilities and lower overall capital costs [15]. Specifically, estimates from economic models show that plants can significantly decrease the cost of biologics as a whole, potentially reducing the cost of mAbs by as much as 50% [49,50]. Under the conditions we used, pIL6RmAb accumulated in *N. benthamiana* leaves at a level of 55.95 µg/g FLW which is higher than what was reported for plant-produced mAbs against the spike protein of SARS-CoV-2 using the same expression vector [35]. Since the expression vector was not optimized for antibody expression in plants, the accumulation levels of pIL6RmAb can be increased significantly by using new versions of expression vectors [36] and/or co-expression with chaperons [51]. It has been shown that under optimal conditions including using improved geminiviral vectors, mAbs can accumulate up to 0.8–4.8 mg/g FLW, a level feasible for commercial mAb production [29,36,52]. pIL6RmAb was also readily purified from plants with a downstream process that has been shown to be scalable with sufficient product recovery and compliant to current Good Manufacturing Practice (cGMP) regulations [28,29,53]. The clinical development of plant-made antibodies against Ebola (ZMapp) and HIV (2G12) along the with the approval of the first plant-made biologic (Taliglucerase alfa) by the US food and drug administration (FDA) not only demonstrates the safety of plant-made mAbs in humans, but also has cleared the regulatory pathway for approving future plant-derived mAb drugs [20,54,55]. pIL6RmAb was produced in a glycoengineered *N. benthamiana* plant line [56,57] which produces mAbs with a homogenous human N-glycosylation form [58]. This eliminates the concern for immunogenicity and the potential risk of adverse effects associated with plant-specific glycans on mAbs and will facilitate the future application of pIL6RmAb in humans. Thus, as anti-IL-6R interventions are continuing to be assessed for COVID-19, there is a potential opportunity for plant-based production of these expensive mAb treatments to ease the economic burden of treating hospitalized patients. In addition, pIL6RmAb may also help to address the high-cost issues of therapeutics for treating RA and other autoimmune diseases as it retains the IL-6 blocker activity of sarilumab.

Almost two years since the SARS-CoV-2 pandemic began, there is still a need for the development of therapeutics, both anti-viral and immunomodulatory, to control the waves of severe infection that are still observed. In this study, we highlight the potential for a plant-made anti-IL-6R mAb to be used as a therapeutic, with specific emphasis to the cytokine release syndrome observed in some critically ill COVID-19 patients [59]. Specifically, we show that the pIL6RmAb can be expressed in plants, that it efficiently blocks IL-6 signaling by binding to the IL-6R, and that this mechanism is preserved in a simulated disease state, In vitro. However, the approved indication of RA for sarilumab still remains an expensive treatment option for many individuals [60]. Along with plant-produced mAbs against the S protein [35,61], plant-produced anti-IL-6R mAb and mAbs against that of other cytokines may provide a less-expensive, yet equally potent treatment option for COVID-19 and other inflammatory diseases.

## Data Availability

The data presented in this study are contained within this article.

## Supplementary Materials

The following are available online at https://www.mdpi.com/article/10.3390/vaccines9111365/s1, Figure S1: Temporal expression pattern of pIL6RmAb in *N. benthamiana* leaves.
