# Supplementary material for: SARS-CoV-2 Spike Protein-Induced Interleukin 6 Signaling Is Blocked by a Plant-Produced Anti-Interleukin 6 Receptor Monoclonal Antibody
Source: Vaccines (Basel). 2021 Nov 20;9(11):1365. doi: 10.3390/vaccines9111365 (PMC8623585; doi:10.3390/vaccines9111365)
Supplement: Supplementary file 1 [file vaccines-09-01365-s001.zip › vaccines-1431886-supplementary.pdf]

Supplemental material

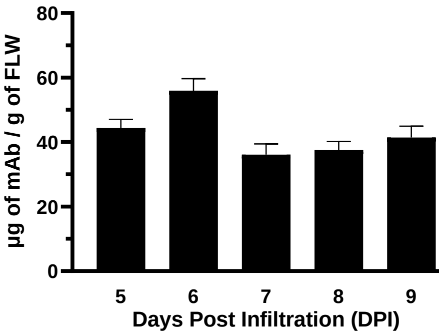

Figure S1. Temporal expression pattern of pIL6RmAb in *N. benthamiana* leaves. Total soluble proteins from pIL6RmAb construct-infiltrated plants were extracted on 5-9 days post infiltration (DPI) and analyzed by an ELISA that detects the assembled form of pIL6RmAb. Mean ± SEM of samples from two independent experiments are presented.
